# Highly rearranged mitochondrial genome in *Falcolipeurus* lice (Phthiraptera: Philopteridae) from endangered eagles

**DOI:** 10.1186/s13071-021-04776-5

**Published:** 2021-05-20

**Authors:** Yu Nie, Yi-Tian Fu, Yu Zhang, Yuan-Ping Deng, Wei Wang, Ya Tu, Guo-Hua Liu

**Affiliations:** 1 Hunan Provincial Key Laboratory of Protein Engineering in Animal Vaccines, College of Veterinary Medicine, Hunan Agricultural University, Changsha, 410128 Hunan China; 2 School of Science and Engineering, GeneCology Research Centre, Animal Research Centre, University of the Sunshine Coast, Sippy Downs, QLD 4556 Australia; 3 Beijing Wildlife Rescue and Rehabilitation Center, Beijing, 101300 China

**Keywords:** Bird lice, Mitochondrial genome, Gene rearrangement, Phylogenetic analyses

## Abstract

**Background:**

Fragmented mitochondrial (mt) genomes and extensive mt gene rearrangements have been frequently reported from parasitic lice (Insecta: Phthiraptera). However, relatively little is known about the mt genomes from the family Philopteridae, the most species-rich family within the suborder Ischnocera.

**Methods:**

Herein, we use next-generation sequencing to decode the mt genome of *Falcolipeurus suturalis* and compare it with the mt genome of *F. quadripustulatus*. Phylogenetic relationships within the family Philopteridae were inferred from the concatenated 13 protein-coding genes of the two *Falcolipeurus* lice and members of the family Philopteridae using Bayesian inference (BI) and maximum likelihood (ML) methods.

**Results:**

The complete mt genome of *F. suturalis* is a circular, double-stranded DNA molecule 16,659bp in size that contains 13 protein-coding genes, 22 transfer RNA genes, two ribosomal RNA genes, and three non-coding regions. The gene order of the *F. suturalis* mt genome is rearranged relative to that of *F. quadripustulatus*, and is radically different from both other louse species and the putative ancestral insect. Phylogenetic analyses revealed clear genetic distinctiveness between *F. suturalis* and *F. quadripustulatus* (Bayesian posterior probabilities=1.0 and bootstrapping frequencies=100), and that the genus *Falcolipeurus* is sister to the genus *Ibidoecus* (Bayesian posterior probabilities=1.0 and bootstrapping frequencies=100).

**Conclusions:**

These datasets help to better understand gene rearrangements in lice and the phylogenetic position of *Falcolipeurus* and provide useful genetic markers for systematic studies of bird lice.

**Graphic abstract:**

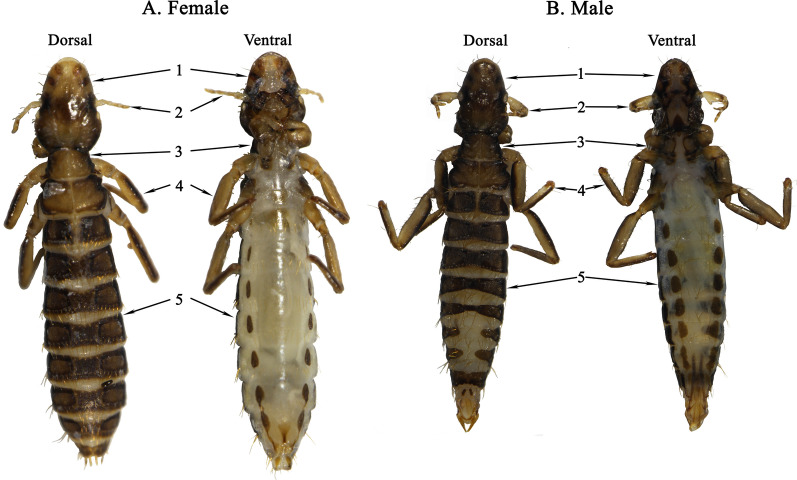

**Supplementary Information:**

The online version contains supplementary material available at 10.1186/s13071-021-04776-5.

## Background

The typical insect mitochondrial (mt) genome is a circular, double-stranded DNA molecule of about 1220kb in length that contains 37 genes: 13 protein-coding genes (PCGs), 22 transfer RNAs (tRNA), and two ribosomal RNAs (rRNA) [1, 2]. However, some lineages of parasitic lice (Insecta: Phthiraptera) are notable exceptions. For example, several groups show extensively fragmented mt genomes, where the genes are separated onto multiple circular chromosomes, including in the families Haematopinidae [3], Hoplopleuridae [4, 5], Menoponidae [6], Pediculidae [7,9], Polyplacidae [10], and Trichodectidae [11]. Parasitic lice are currently divided into four suborders, namely Ischnocera, Amblycera, Rhynchophthirina, and Anoplura, based on morphological structure.

Lice are permanent, obligate, and often host-specific ectoparasites commonly found on birds and mammals. The suborder Ischnocera (approximately 3120 species) is currently divided into two families, Philopteridae (approximately 2600 species) and Trichodectidae [12]. Complete mt genomes of only a limited number of philopterid species have been sequenced: *Bothriometopus macrocnemis* [13], *Campanulotes bidentatus compar* [14], *Campanulotes compar* [11], *Coloceras* sp. [15], *Falcolipeurus quadripustulatus* [11], *Ibidoecus bisignatus* [15], *Columbina picui*, *Columbina cruziana*, and *Columbicola columbae* [16]. These studies have found extensive gene rearrangements in philopterid mt genomes. A recent report has demonstrated that highly fragmented, mt minicircles are present in four species of the genus *Columbicola* [16], indicating that fragmented mt genomes are more prevalent in parasitic lice than previously hypothesized [11]. These studies demonstrate that our knowledge of the structure in mt genomes of bird lice is far from comprehensive. Additional data is needed to understand the pattern and mechanisms of genome fragmentation and rearrangement in bird lice.

Owing to maternal inheritance, relatively high evolution rate, conserved gene components, and low rate of recombination, the mt genome has been widely used as a genetic marker for comparative, evolutionary, and phylogenetic analysis at different taxonomic levels [17, 18]. In this study, we (i) characterize the complete mt genome sequences of *F. suturalis* from the tawny eagle, (ii) compare it with that of *F. quadripustulatus* from the vulture, and (iii) assess the phylogenetic position of *Falcolipeurus* within the Philopteridae.

## Methods

### Sample collection and DNA extraction

**Fig. 1:**
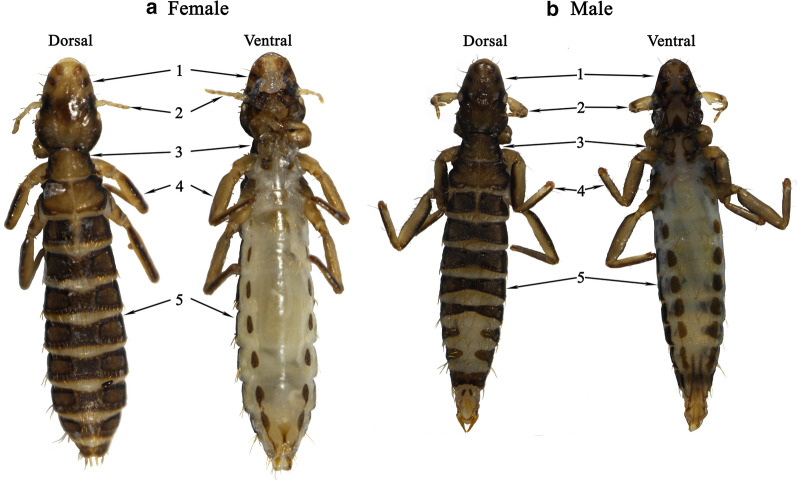
Female (**a**) and male (**b**) *Falcolipeurus suturalis* (dorsal side and ventral side). 1: head, 2: antenna, 3: thorax, 4: legs, 5: abdomen

Adult samples of *F. suturalis* were taken from a tawny eagle *Aquila rapax* in the Beijing Wildlife Rescue Center, China. Lice were washed twice with sterile physiological saline solution (0.85%), and initial identification as *F. suturalis* made based on morphology and host species (Fig.1) [19], and then stored in 95% (v/v) ethanol at 40C. Total genomic DNA was extracted from 60 individual lice (30 females and 30 males) using the DNeasy Tissue Kit (Promega, Madison, USA) following manufacturer instructions. Two pairs of primers [20], mtd6-mtd11 and 12SA-12SB, were used to amplify fragments of *cox*1 (600bp) and *rrn*S (350bp) genes, respectively, for use as assembly baits [4].

### Sequencing, assembling and verification

The quality of extracted genomic DNA was tested by agarose gel electrophoresis [21] and DNA concentration were quantified by Qubit 2.0 Fluorometer (Thermo Scientific). A genomic DNA library (350bp inserts) was prepared and sequenced by Novogene Bioinformatics Technology Co., Ltd. (Tianjin, China) using Illumina HiSeq 2500 (250bp paired-end reads). Raw reads were filtered with PRINSEQ [22]. Preliminary *cox*1 and *rrn*S sequences of *F. suturalis* were used as initial references for assembly in Geneious v 11.1.5 (minimum overlap identity=99%, minimum overlap=150bp, maximal gap size=5bp) [23]. Genome size and circular organization were verified by long PCR using four pairs of specific primers (Additional file 1: Table S1; Additional file 2: Figure S1).

### Annotation and sequence analysis

Protein-coding and rRNA genes were identified by alignment to homologous genes of previously sequenced mt genome of the vulture louse *F. quadripustulatus* [11] using the MAFFT v7.122 software [24]. tRNA genes were identified using ARWEN [25] and tRNAscan-SE [26], with manual adjustment. Annotated mt genomes were illustrated using the visualize module of MitoZ [27]. Nucleotide composition and amino acid sequences of each protein-coding genes and codon usage were analyzed using MEGA v6.0 [28]. Asymmetry of base composition was calculated as the following formula: AT-skew=(*AT*)/(*A*+*T*), GC-skew=(*GC*)/(*G*+*C*) [29].

### Phylogenetic analysis

A total of 23 mt genomes from lice, including ten species from the Philopteridae, three species from the Trichodectidae, one elephant louse, *Haematomyzus elephantis*, and eight species of sucking lice, were used for phylogenetic analysis [3, 5, 7, 9,16, 20, 30,32], with one wallaby louse, *Heterodoxus macropus* (GenBank: AF270939), used as an outgroup [33]. Amino acid sequences of 12 protein-coding genes (except for *nad*2 because it is unidentified in the *H. elephantis* mt genome) were aligned individually using MAFFT [23]. Alignments of the individual genes were concatenated into a single dataset. Ambiguously aligned areas were removed by Gblocks 0.91b with the option of less stringent selection [34], and subjected to phylogenetic analyses under Bayesian inference (BI) and maximum likelihood (ML). BI was conducted with four independent Markov chains run for 5,000,000 metropolis-coupled MCMC generations, sampling trees every 500 generations in MrBayes v3.2.7 [35]. The initial 25% (2500) trees of each MCMC were discarded as burn-in and the majority-rule consensus tree used to calculate Bayesian posterior probabilities (Bpp). For ML analysis, the alignment was partitioned by gene, and bootstrapping frequencies (Bf) performed using the rapid bootstrapping option with 100 iterations. The MtART (all 12 genes) model was selected as the most suitable model of evolution by ProtTest v2.4 based on the Akaike information criterion (AIC) [36]. ML analyses were computed using RAxML v2.2.3 [37]. Phylograms were drawn using FigTree v.1.31.

## Results and discussion

### Identity of the eagle louse *F. suturalis*

Seven louse species (*F. suturalis*, *Degeeriella fulva*, *Degeeriella aquilarum*, *Nosopon chanabense*, *Colpocephalum impressum*, *Laemobothrion Laemobothrion vulturis*, and *Laemobothrion Laemobothrion maximum*) can parasitize the tawny eagle *A. rapax* (http://phthiraptera.info/category/avian/aves/falconiformes/accipitridae/aquila/aquila-rapax).

*F. suturalis* can be identified by the following morphological characters: (1) body slender in deep dark brown colour; (2) head longer than wide with pointed anterior and slightly broad posterior; (3) dorsally, 3 dark brown spots on each side of preantennal area and 1 dark brown horizontal stripe near antennal area; (4) antennae sexually dimorphic, male antennae stout and long on segment 1, female antenna slender and short on all 5 segments; (5) thorax widened from top to bottom; (6) dorsal thorax with 1 pronotum and 2 pteronotums, pronotum larger than pteronotum, 1 stout short seta on posterior margin of each pteronotum; (7) ventral thorax, mesosternum with 1 pair dark brown spots near midlegs; (8) 3 pairs of legs slender, forelegs small, midlegs and hindlegs progressively larger; (9) abdomen longer than wide; (10) dorsal abdomen deep dark brown colour, 2nd segment slightly narrower than thoracic pteronotum and with 2 divided tergite plates; (11) tergites on dorsal abdominal segments 3 to 6 slightly narrow in middle on both male and female; (12) male with 2 divided tergite plates laterally and female with 1 tergite on each dorsal abdominal segment of 7 and 8; (13) ventral abdomen with 9 segments and in white colour, 1 pair of dark brown spots laterally on each segment of 2 to 9; (14) female with 1 small dark brown spot in inverted triangle shape in middle of ventral abdominal segments 8 and 9.

### Genome organization

A total of 3.7Gb of data (about 20-fold coverage) was obtained from the Illumina HiSeq 2500 platform, raw sequencing of 15,178,382 paired reads. Reads were cleaned (9,630,532 clean pairs) and assembled. The longest assembled contig was 16,659bp in size and was the complete mt genome of *F. suturalis* (GenBank accession: MW696813). All 37 mt genes typical of metazoan mt genomes were present. Gene arrangement was distinct from that of *F. quadripustulatus* [11] and other philopterids [15]. Overall nucleotide composition was: *A*=27.8%, *T*=44.8%, *C*=11.1%, *G*=16.3%. All mt genes were encoded on the heavy strand, except tRNA-Arg. Three overlapping regions in the mt genome were observed: *nad*4L/*nad*1, tRNA-His/tRNA-Asp and tRNA-Asp/tRNA-Arg, ranging from 4 to 8bp overlaps (Table 1). In additional, 22 intergenic regions were observed, ranging from 1 to 180bp in size. The longest spacer was between tRNA-S_2_ and tRNA-G (Table 1).

**Table 1:** The organization of the mt genome of *Falcolipeurus suturalis*

Gene/region	Positions	Strand	Size (bp)	Number of aa^a^	Ini/Ter codons^b^	Anticodon	In^c^
*cox*1	341557	H	1524	507	ATA/TAA		+33
tRNA-Met (M)	15741637	H	64			CAT	+16
tRNA-Gln (Q)	16391705	H	67			TTG	+1
tRNA-Glu (E)	17061770	H	65			TTC	0
*atp*6	17742445	H	672	223	ATA/TAA		+3
tRNA-Asn (N)	24512517	H	67			GTT	+5
*rrn*S	25183243	H	726				0
*rrn*L	32444318	H	1075				0
tRNA-Ala (A)	43194382	H	64			TGC	0
*nad*6	43854858	H	474	157	ATG/TAA		+2
tRNA-Val (V)	48614922	H	62			TAC	+2
*cox*3	49775726	H	750	249	ATA/TAA		+54
tRNA-Lys (K)	57465808	H	63			TTT	+19
*nad*4	58437156	H	1314	437	ATT/TAG		+34
NCR1	71577985	H	829				
tRNA-Leu^UUR^ (L_2_)	79868047	H	62			TAA	0
tRNA-Pro (P)	80648124	H	61			TGG	+16
*nad*2	81309101	H	972	323	ATG/TAA		+5
tRNA-Thr (T)	91719235	H	65			TGT	+69
tRNA-Tyr (Y)	92499313	H	65			GTA	+13
*cox*2	93149991	H	678	225	ATA/TAA		0
NCR2	999210,713	H	722				
*nad*5	10,71412,389	H	1676	558	ATG/TA		0
tRNA-Phe (F)	12,39012,456	H	67			GAA	0
tRNA-Cys (C)	12,47712,543	H	67			GCA	+20
*atp*8	12,56512,765	H	201	66	ATG/TAA		+21
tRNA-Ser^UCN^ (S_2_)	12,77212,840	H	69			TGA	+6
tRNA-Gly (G)	13,02113,091	H	71			TCC	+180
NCR3	13,09213,516	H	425				
*nad*3	13,51713,903	H	387	128	ATT/TAG		0
tRNA-Leu^CUN^ (L_1_)	13,90513,966	H	62			TAG	+1
*nad*4L	13,99214,264	H	273	90	ATT/TAA		+25
*nad*1	14,25715,163	H	907	302	ATG/T		8
tRNA-Ser^AGN^ (S_1_)	15,16415,231	H	68			TCT	0
*cyt*b	15,23216,323	H	1092	363	TTG/TAG		0
tRNA-Trp (W)	16,33016,396	H	67			TCA	+6
tRNA-His (H)	16,39816,460	H	63			GTG	+1
tRNA-Asp (D)	16,45716,524	H	68			GTC	4
tRNA-Arg (R)	16,58516,516	L	70			ACG	8
tRNA-Ile (I)	16,59316,659	H	67			GAT	+6

^a^The inferred length of amino acid (aa) sequence of 13 protein-coding genes

^b^Ini/Ter codons: initiation and termination codons

^c^In: Intergenic nucleotides

Total *A*+*T* and *G*+*C* content of the complete mt genome was 73.0% and 27.0%, respectively, consistent with the nucleotide content of lice reported in previous studies [11, 15] (Table 2). Negative AT skew (23.3) and positive GC skew (18.9) were found (Table 2), consistent with other louse mt genomes [11, 15]. All bird lice from the Philopteridae reported to date have strong strand asymmetry (GC skew between 6.3% and 38.1%) (Table 2).

**Table 2:** Nucleotide composition of the mt genomes of Philopteridae species, including that of *Falcolipeurus suturalis*

Species	Nucleotide frequency (%)				Whole genome sequence		
	*A*	*T*	*G*	*C*	*A*+*T*%	AT skew	GC skew
*Bothriometopus macrocnemis*	32.1	38.7	15.5	13.8	70.8	9.2	6.1
*Campanulotes bidentatus compar*	26.5	43.7	20.67	9.77	70.1	24.5	38.1
*Campanulotes compar*	26	44.5	20.4	9.1	70.5	26.3	38.1
*Coloceras* sp.	27.5	42.9	19.9	9.6	70.4	21.8	35.1
*Ibidoecus bisignatus*	35.5	40.6	13.2	10.8	76	6.7	10.2
*Columbicola columbae*	39.1	29.2	16.3	15.4	68.2	14.6	2.8
*Columbina picui*	33.5	31.6	18.3	16.6	65.1	2.9	5
*Columbina cruziana*	32.9	31.4	19	16.7	64.3	2.4	6.3
*Falcolipeurus quadripustulatus*	26.3	45.5	16.9	11.3	71.8	26.8	20.1
*Falcolipeurus suturalis*	28	45	16.4	11.2	73	23.3	18.9

### Annotation

**Fig. 2:**
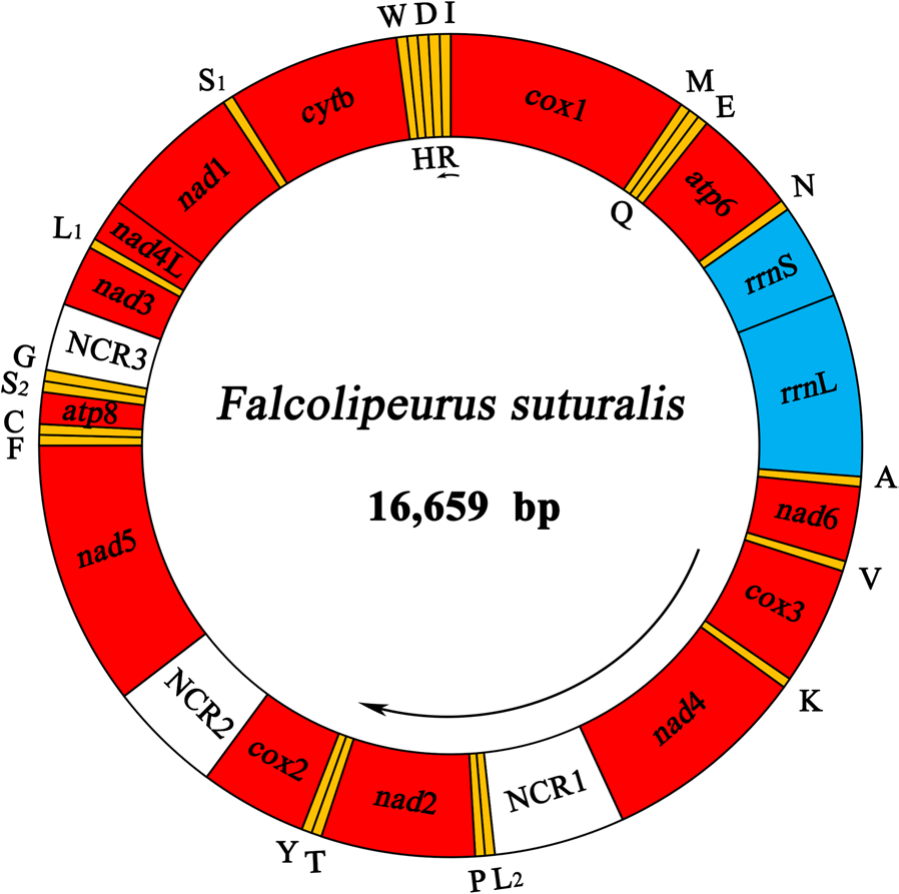
The mt genome of *Falcolipeurus suturalis*. All genes are on the same DNA strand and are transcribed clockwise, except for tRNA-Arg (R). Protein-coding and rRNA genes are indicated with the standard nomenclature. tRNA genes are indicated with the one-letter code of their corresponding amino acids. There are two tRNA genes for leucine: L_1_ for codons CUN and L_2_ for UUR; and two tRNA genes for serine: S_1_ for codons AGN and S_2_ for UCN. NCR1 refers to the first non-coding region. NCR2 refers to the second non-coding region. NCR3 refers to the third non-coding region

**Fig. 3:**
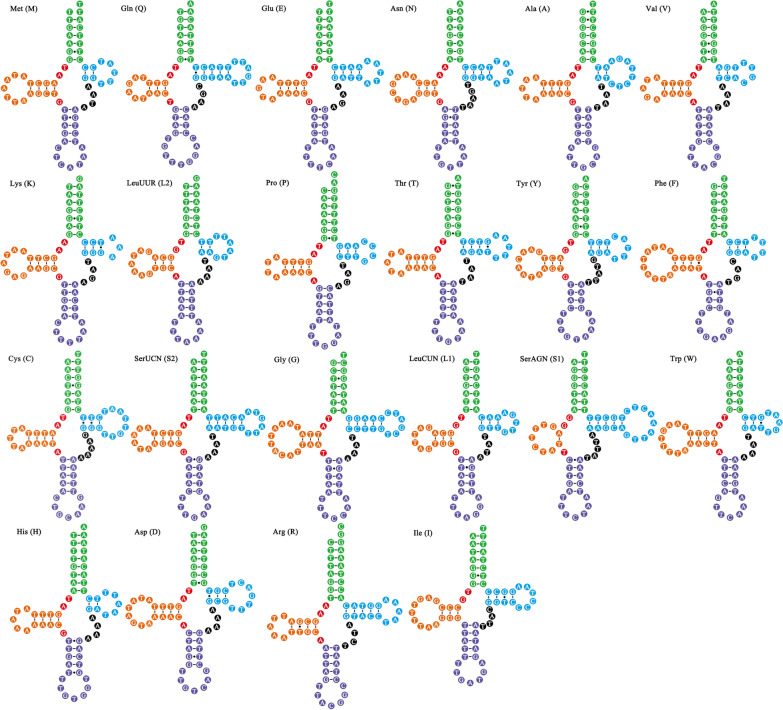
22 tRNA secondary structures from *Falcolipeurus suturalis*

As the mt genomes of parasitic lice can contain non-standard initiation codons [1, 5, 13], their identification can be challenging. In *F. suturalis*, all protein-coding genes used ATNs (ATA, ATG, ATT) or TTG as their initiation codons. Four genes (*cox*1, *atp*6, *cox*3, and *cox*2) use ATA, five (*nad*6, *nad*2, *nad*5, *atp*8, and *nad*1) use ATG, three (*nad*3, *nad*4L, and *nad*4) use ATT, and one (*cyt*b) uses TTG (Table 1). All protein-coding genes had standard (TAA or TAG) or partial (TA or T) termination codons (Table 1). Eight genes (*cox*1, *atp*6, *nad*6, *cox*3, *nad*2, *cox*2, *atp*8, and *nad*4L) use TAA, three genes (*nad*4, *nad*3, and *cyt*b) use TAG, *nad*1 uses TA, and *nad*5 genes use T (Table 1). Incomplete termination codons (TA or T) were found for *nad*1 and *nad*5 genes, which has been found in other bird lice, including *B. macrocnemis* (*nad*1) and *F. quadripustulatus* (*nad*5, *nad*6 and *nad*1). In the *F. suturalis* mt genome, *rrn*L was located between *rrn*S and tRNA-Ala, while *rrn*S was between tRNA-Asn and *rrn*L (Fig.2; Table 1). The lengths of *rrn*S and *rrn*L were 726bp and 1075bp, respectively. tRNA genes varied from 61 to 71bp in length (Table 1). All 22 tRNA genes can be folded into the canonical cloverleaf structure (Fig.3), consistent with previous studies [31, 32]. Apart from the coding regions, we identified three non-coding regions. Non-coding region (NC1) (829bp), located between *nad*4 and tRNA-Leu^UUR^, has the highest *A*+*T* content of 75.4% while non-coding region (NC2) (722bp; *A*+*T*=74.4%) is located between *cox*2 and *nad*5 and non-coding region (NC3) (425bp; *A*+*T*=75.1%) is located between tRNA-Gly and *nad*3 (Table 1).

### Comparative analyses between *F. suturalis *and *F. quadripustulatus*

The entire mt genome of *F. suturalis* is 537bp longer than that of *F. quadripustulatus* [11]. A comparison of nucleotide and amino acid sequences in each protein-coding gene of the two *Falcolipeurus* species is given in Table 3. Nucleotide sequence differences across the entire mt genome was 31.4%. The magnitude of nucleotide sequence variation in each gene between *F. suturalis* and *F. quadripustulatus* ranged from 13.2 to 27.5%. The greatest variation was observed in *atp*8 (27.5%), and the least difference was found in *cyt*b (13.2%) (Table 3). For *rrn*L and *rrn*S, sequence difference is 28.4% and 14.6%, respectively, between *F. suturalis* and *F. quadripustulatus* (Table3). Amino acid sequences inferred from individual mt protein genes of *F. suturalis* were also compared with those of *F. quadripustulatus*. Amino acid sequence differences ranged from 4.5 to 41.2%, with *cox*1 being the most conserved protein, and *atp*8 the least conserved (Table 3). Our results are consistent with other species-level comparisons in lice. For example, amino acid divergence in the 13 protein-coding genes of *C. picui* and *C. cruziana* was 5.550% [16], while between *C. bidentatus compar* and *C. compar* it was 037.3% [11, 14]. Taken together, the molecular evidence presented here supports that *F. suturalis* and *F. quadripustulatus* represent distinct louse species.

**Table 3:** Nucleotide (nt) and/or predicted amino acid (aa) sequence differences in mitochondrial genes among *Falcolipeurus quadripustulatus* (FQ) and *Falcolipeurus suturalis* (FS) upon pairwise comparison

Gene/region	Nt sequence length		Nt difference (%)	Number of aa		aa difference (%)
	FS	FQ	FS/FQ	FS	FQ	FS/FQ
*cox*1	1524	1554	15.3	507	517	4.5
*atp*6	672	675	18.5	223	224	16.1
*rrn*S	726	610	28.4			
*rrn*L	1075	1084	14.6			
*nad*6	474	478	22	157	159	25.8
*cox*3	750	789	21.2	249	265	16.2
*nad*4	1314	1305	24	437	434	27.2
*nad*2	972	972	27	323	323	32.5
*cox*2	678	675	14.4	225	224	7.5
*nad*5	1676	1711	19.6	558	570	21
*atp*8	201	204	27.5	66	67	41.2
*nad*3	387	354	27.4	128	117	34.4
*nad*4L	273	288	20.8	90	95	21.1
*nad*1	907	848	20	302	282	12.6
*cyt*b	1092	1092	13.2	363	363	9

### Gene rearrangement

**Fig. 4:**
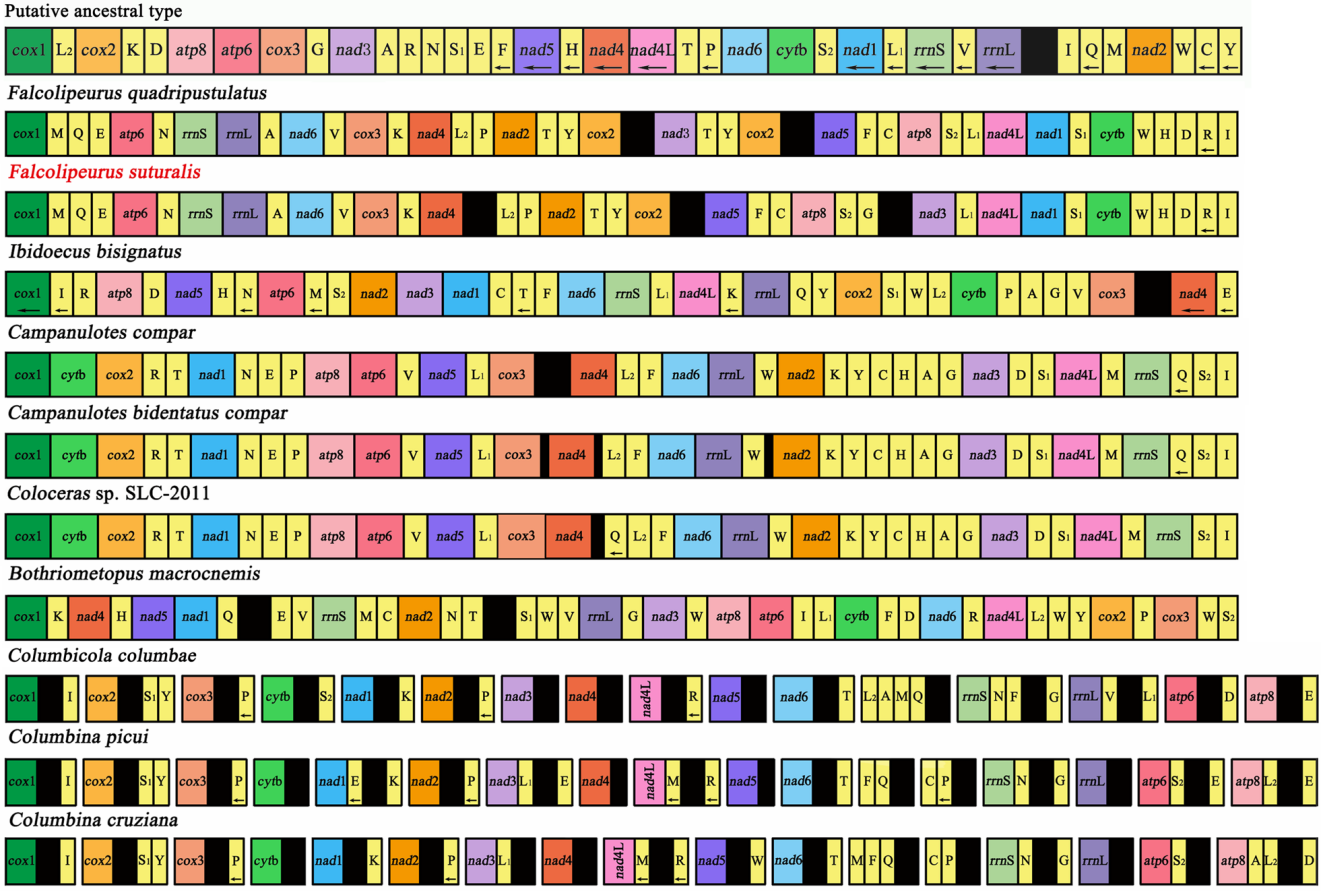
Gene rearrangements of mitochondrial genomes of bird lice within the family Philopteridae

The mt genome arrangements of both *Falcolipeurus* species differ substantially from those of other species in the Philopteridae and from the putative ancestral insect (Fig.4). Compared with the putative ancestral insect, no mt gene arrangements are shared with the mt genomes of *Falcolipeurus* species, as all genes are moved and/or inverted relative to their ancestral positions (Fig.4). Of the 13 protein-coding genes, four (*nad*5, *nad*4, *nad*4L, and *nad*1) are inverted in both *Falcolipeurus* species relative to the putative ancestral insect. Only two gene blocks are shared between *Bothriometopus* and the putative ancestral insect: tRNA-Gly*nad*3 and *atp*8*atp*6 [13], while one gene block is shared between *Campanulotes* species and the ancestral insect: *atp*8-*atp*6 [11, 14, 33]. Three gene blocks, tRNA-Val*cox*3, tRNA-Tyr*cox*2, and tRNA-Leu^CUN^*nad*4L, are shared between *Falcolipeurus* and *Ibidoecus* [11]. Two gene blocks, *nad*4tRNA-Leu^UUR^ and tRNA-Gly*nad*3, are shared between *Falcolipeurus* and *Campanulotes*. In the Philopteridae, only one gene block, tRNA-Ile*cox*1, is shared across all Philopterids, excepting *I. bisignatus, Columbina*, and *Columbicola*. Such a lack of conserved gene arrangements in the mt genome of bird lice complicates the accurate reconstruction and identification of rearrangement events across their history [13].

**Fig. 5:**
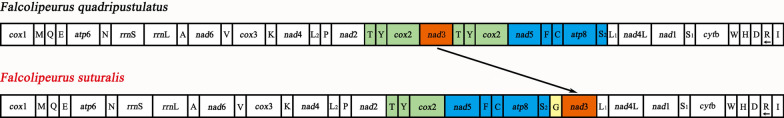
Gene rearrangement in two *Falcolipeurus* species

Usually, gene arrangement in mt genomes is very conserved between congeneric lice [11, 14]. Gene rearrangements between *F. suturalis* and *F. quadripustulatus* were identified consisting of at least one translocation (Fig.5). The *nad*3 gene is located between *cox*2 and tRNA-Thr in *F. quadripustulatus*, but between tRNA-Gly and tRNA-Leu^CUN^ in *F. suturalis* (Fig.5). This gene rearrangement between the mt genomes of two *Falcolipeurus* species indicated that the rate of rearrangements of mt genes may vary substantially among closely related groups of lice [38]. It is interesting that two congeneric *Falcolipeurus* species differ by a rearrangement. Although this pattern has been found in *Columbicola* [16] and multiple Anoplura species [11], it is the first time that it has been found in a louse without fragmented genomes.

One tRNA gene (tRNA-Gly) was lacking, while the duplication of three genes (tRNA-Thr, tRNA-Tyr, and *cox*2) was found in the *F. quadripustulatus* mt genome [11]. However, all 37 genes have been identified in the *F. suturalis* mt genome. Gene duplications have also been reported in the mt genomes of several families in the class Insecta, such as the Reduviidae and Thripidae [39,41]. In addition, tRNA loss has been also found in the mt genome of several families of the class Insecta [11, 42].

### Phylogenetic relationships

**Fig. 6:**
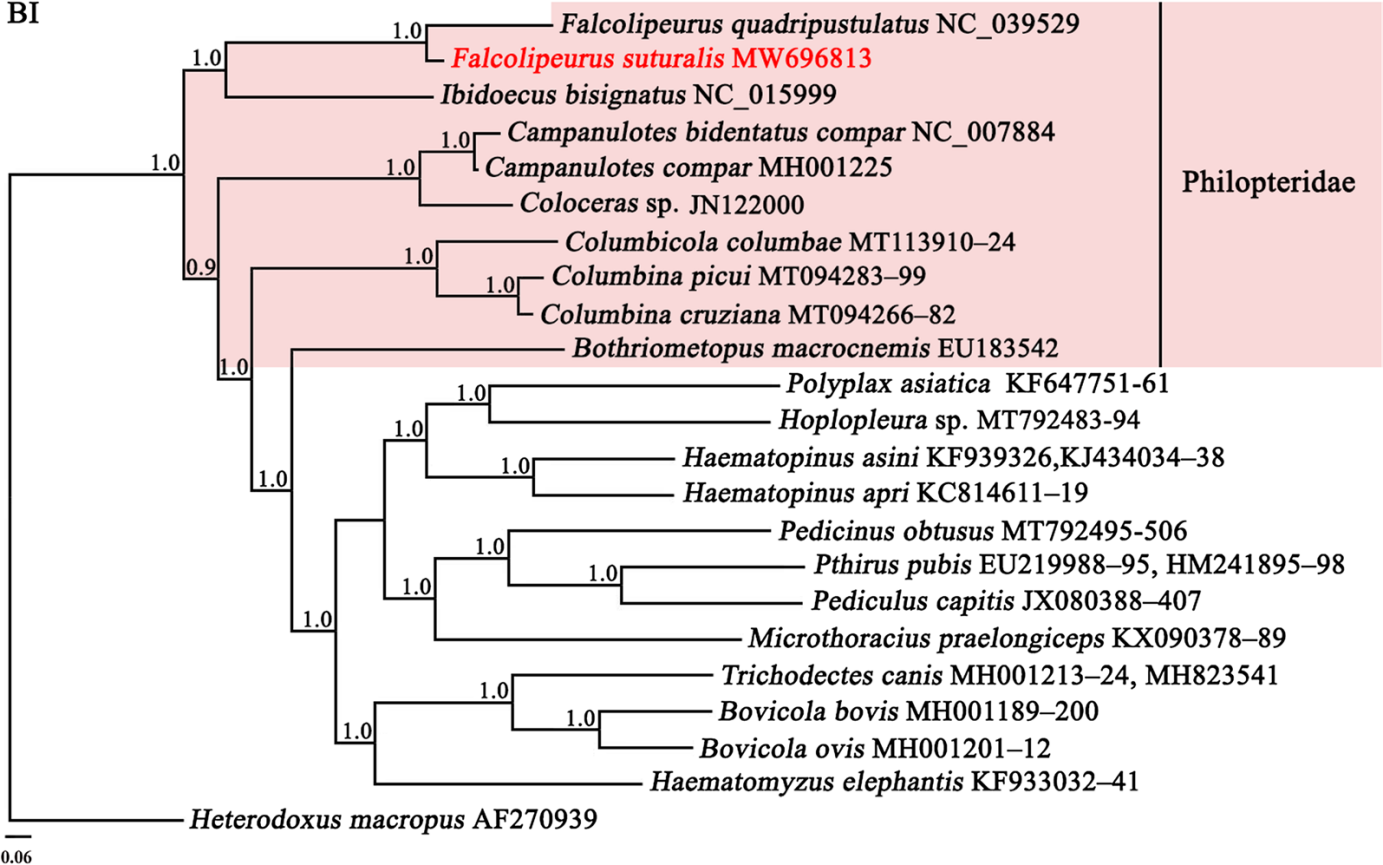
Phylogenetic relationships among 10 species of the family Philopteridae inferred by Bayesian inference from deduced amino acid sequences of 12 protein-coding genes. One wallaby louse, *Heterodoxus macropus* as an outgroup. Bayesian posterior probabilities (Bpp) were indicated at nodes

**Fig. 7:**
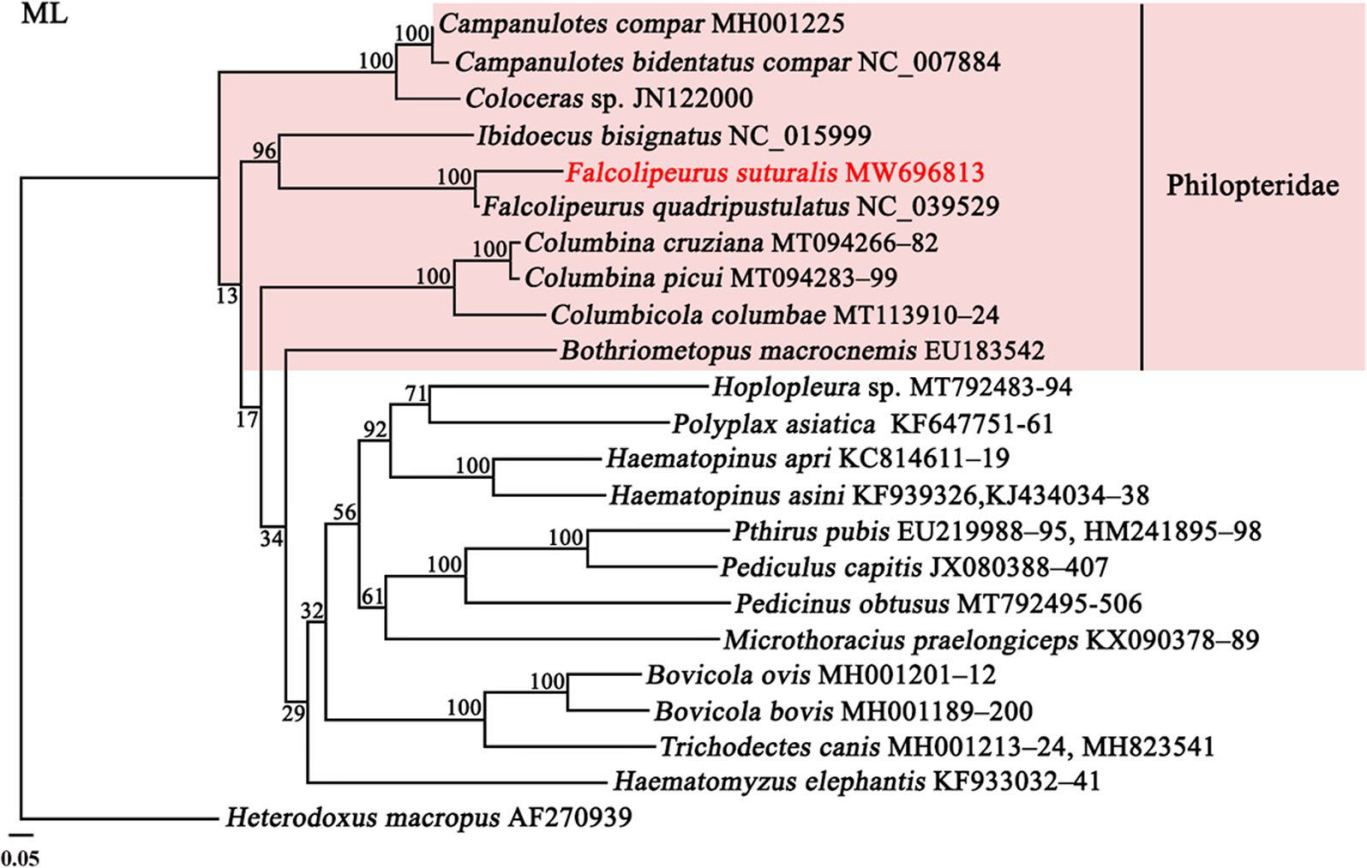
Phylogenetic relationships among 10 species of the family Philopteridae inferred by Maximum likelihood from deduced amino acid sequences of 12 protein-coding genes. One wallaby louse, *Heterodoxus macropus* as an outgroup. Bootstrapping frequencies (Bf) were indicated at nodes

Phylogenetic analysis shows the genetic distinctiveness between *F. suturalis* and *F. quadripustulatus* (Bpp=1.0; Bf=100). The branch leading to the two *Falcolipeurus* species is much longer than the branch of two *Columbina* species. The genus *Falcolipeurus* is more closely related to the genus *Ibidoecus* than to other genera (Bpp=1.0, Fig.6; Bf=100, Fig.7), which was consistent with that of a previous study [11]. Ten species of the Philopteridae were included in this study. The Philopteridae was paraphyletic with strong support in Bayesian analysis (Bpp>0.9, Fig.6) and weak support in ML analysis (Bf>17, Fig.7).

DNA sequencing provides the opportunity to further evaluate phylogenetic relationships in the Philopteridae. Phylogenetic relationships in the Philopteridae have been investigated with analyses of the nuclear gene sequences. For example, Cruickshank et al. analyzed elongation factor-1 alpha (EF-1) sequences of 127 species from the four louse suborders, showing the Philopteridae to be paraphyletic [43]. Yoshizawa and Johnson analyzed mt 12S and 16S rDNA sequences of 18 species also showed the family to be paraphyletic [44]. However, Johnson et al. analyzed 1107 single-copy orthologous genes of 46 species and showed that the Philopteridae was monophyletic [45]. De Moya et al. analyzed 2370 orthologous genes and also showed that Philopteridae was monophyletic [46].

Mt genome sequences are effective molecular markers for the study of phylogenetic and systematic relationships at various taxonomic ranks across the phylum Arthropoda, including for ectoparasites [44,48]. Recently, a study has also indicted that mt genes can provide reliable reconstructions of evolutionary relationships in parasitic lice [6]. In the present study, the monophyly of the Philopteridae was rejected by mt genomic phylogenetic analyses. Song et al. inferred the high-level phylogeny of parasitic lice with the mt genome sequences of 25 species of parasitic lice, and showed that the Philopteridae was paraphyletic [11]. To date, the phylogenetic position of the Philopteridae within the Phthiraptera has not been confidently determined. Although mt genomic data has proven to be useful as genetic markers to explore the phylogenetic relationships among major lineages of parasitic lice [9, 11], mt genome sequences of many lineages are under- or not represented. Analyses of mt genome sequences in the current and previous studies has indicated that the Philopteridae was paraphyletic. However, many species of this family were not included across these studies [11]. Additional complete mt genomes of bird louse species representing multiple families should be included in future analysis to help resolve the phylogenetic position of the Philopteridae within the Phthiraptera.

## Conclusions

The current study presents the entire mt genome of *F. suturalis* and compared it with the mt genome of *F. quadripustulatus*. Its gene order is rearranged relative to other *Falcolipeurus* species, and represents a new pattern within the Phthiraptera. These novel datasets will help to better understand the gene rearrangements and phylogenetic position of *Falcolipeurus* and provide useful genetic markers for systematics and phylogenetic studies of bird lice.

## Supplementary Information

**Additional file 1: Table S1.** Primers used for assembly validation.

**Additional file 2: Figure S1.** PCR amplicons from the mitochondrial genome of *Falcolipeurus suturalis*. Amplicons generated with the *F. suturalis* primers. M: DL8000 DNA marker, 1: Validation_01, 2: Validation_02, 3: Validation_03, 4: Validation_04, 5: Validation_05, 6: Negative control.

## Data Availability

The complete mitochondrial genome sequences of *Falcolipeurus suturalis* have been deposited in the GenBank database under the accession number MW696813.

## Abbreviations

mt: Mitochondrial
rDNA: Ribosomal DNA
BI: Bayesian inference
*nad*2: NADH dehydrogenase subunit 2
*cox*1: Cytochrome c oxidase subunit 1
*cox*2: Cytochrome c oxidase subunit 2
*atp*6: ATP synthase F0 subunit 6
*cox*3: Cytochrome c oxidase subunit 3
*nad*3: NADH dehydrogenase subunit 3
*nad*5: NADH dehydrogenase subunit 5
*nad*4: NADH dehydrogenase subunit 4
*nad*4L: NADH dehydrogenase subunit 4L
*nad*6: NADH dehydrogenase subunit 6
*cyt*b: Cytochrome b
*atp*8: ATP synthase F0 subunit 8
*nad*1: NADH dehydrogenase subunit 1
tRNA: Transfer RNA
*rrn*L: Large subunit of rRNA
*rrn*S: Small subunit of rRNA
BI: Bayesian inference
ML: Maximum likelihood
AIC: Akaike information criterion
Bpp: Bayesian posterior probabilities
Bf: Bootstrapping frequencies
