# Supplementary material for: Highly rearranged mitochondrial genome in Falcolipeurus lice (Phthiraptera: Philopteridae) from endangered eagles
Source: Parasit Vectors. 2021 May 20;14:269. doi: 10.1186/s13071-021-04776-5 (PMC8139141; doi:10.1186/s13071-021-04776-5)
Supplement: Supplementary file 1 — Additional file 1: Table S1. Primers used for assembly validation. [file 13071_2021_4776_MOESM1_ESM.docx]

**Table S1** Primers used for assembly validation.

| Primer | Sequence (5′–3′) | Size of amplified region (bp) |
| --- | --- | --- |
| YZ1F | TAGATGTAGGTGTTGGAACTGGGTGAA | ⁓ 5000 |
| YZ1R | AACCCTATCAGCCATACTAAACCTA |  |
| YZ2F | GCATTTCGTAGATGTAGTATGGTTA | ⁓ 3000 |
| YZ2R | TGACGAGAAGAGGGATAGTAATAAG |  |
| YZ3F | AATCATCTTATTACTATCCCTCTTCTCG | ⁓ 6000 |
| YZ3R | TGTCTAACTGAGATTGAAGGTGGAG |  |
| YZ4F | AAATCTCCACCTTCAATCTCAGTTA | ⁓ 3000 |
| YZ4R | TTCACCCAGTTCCAACACCTACATC |  |
