# Supplementary figures and images for: Highly rearranged mitochondrial genome in Falcolipeurus lice (Phthiraptera: Philopteridae) from endangered eagles
Source: Parasit Vectors. 2021 May 20;14:269. doi: 10.1186/s13071-021-04776-5 (PMC8139141; doi:10.1186/s13071-021-04776-5)

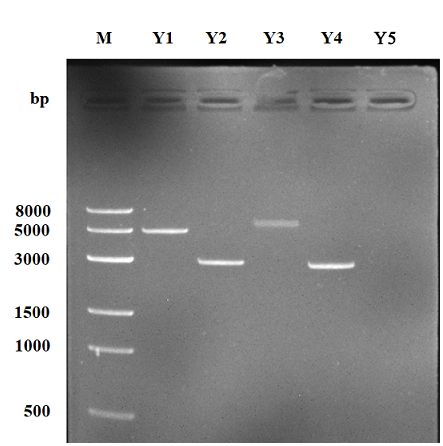

Supplement: Supplementary file 2 — Additional file 2: Figure S1. PCR amplicons from the mitochondrial genome of Falcolipeurus suturalis. Amplicons generated with the F. suturalis primers. M: DL8000 DNA marker, 1: Validation_01, 2: Validation_02, 3: Validation_03, 4: Validation_04, 5: Validation_05, 6: Negative control. [file 13071_2021_4776_MOESM2_ESM.tif]
